# E-NTPDases: Possible Roles on Host-Parasite Interactions and Therapeutic Opportunities

**DOI:** 10.3389/fcimb.2021.769922

**Published:** 2021-11-09

**Authors:** Lisvane Paes-Vieira, André Luiz Gomes-Vieira, José Roberto Meyer-Fernandes

**Affiliations:** ^1^ Laboratório de Bioquímica Celular, Instituto de Bioquímica Médica Leopoldo de Meis, Centro de Ciências da Saúde, Universidade Federal do Rio de Janeiro, Rio de Janeiro, Brazil; ^2^ Departamento de Bioquímica, Instituto de Química, Universidade Federal Rural do Rio de Janeiro, Seropédica, Brazil; ^3^ Instituto Nacional de Ciência e Tecnologia em Biologia Estrutural e Bioimagem, Rio de Janeiro, Brazil

**Keywords:** Ecto-nucleotidases, E-NTPDases, purinergic signaling, parasite infection, antiparasitic drugs

## Abstract

Belonging to the GDA1/CD39 protein superfamily, nucleoside triphosphate diphosphohydrolases (NTPDases) catalyze the hydrolysis of ATP and ADP to the monophosphate form (AMP) and inorganic phosphate (Pi). Several NTPDase isoforms have been described in different cells, from pathogenic organisms to animals and plants. Biochemical characterization of nucleotidases/NTPDases has revealed the existence of isoforms with different specificities regarding divalent cations (such as calcium and magnesium) and substrates. In mammals, NTPDases have been implicated in the regulation of thrombosis and inflammation. In parasites, such as *Trichomonas vaginalis, Trypanosoma* spp.*, Leishmania* spp.*, Schistosoma* spp. and *Toxoplasma gondii*, NTPDases were found on the surface of the cell, and important processes like growth, infectivity, and virulence seem to depend on their activity. For instance, experimental evidence has indicated that parasite NTPDases can regulate the levels of ATP and Adenosine (Ado) of the host cell, leading to the modulation of the host immune response. In this work, we provide a comprehensive review showing the involvement of the nucleotidases/NTPDases in parasites infectivity and virulence, and how inhibition of NTPDases contributes to parasite clearance and the development of new antiparasitic drugs.

## Introduction

There are four important subfamilies of ecto-nucleotidases with different substrate specificities (nucleotide hydrolysis): (i) the ecto-nucleoside triphosphate diphosphohydrolases (E-NTPDases), (ii) the ecto-5’nucleotidases (5’-NT), (iii) the ecto-nucleotidase pyrophosphatase/phosphodiesterases (NPPs), and (iv) alkaline phosphatases (Aps) (Barros et al., 2000; Meyer-Fernandes et al., 2000; de Souza Leite et al., 2007; Paletta-Silva and Meyer-Fernandes, 2012; Zimmermann et al., 2012).

The ecto-nucleoside triphosphate diphosphohydrolases are ecto-nucleotidases that hydrolyze different tri-and diphosphate nucleosides to their monophosphate counterpart (Zimmermann et al., 2012; Zimmermann, 2021a). The E-NTPDase activity is dependent on the presence of divalent cations, such as calcium and magnesium (Handa and Guidotti, 1996; Robson et al., 2006).

Eight members of the E-NTPDase family were identified in mammals. The isoforms 1 (known as CD39), 2, 3, and 8 are typical E-NTPDases, located on the cell surface. The isoforms 4 and 7 were found in the Golgi apparatus and intracellular membrane compartment, respectively. After heterologous expression, the isoforms 5 and 6 were found only in the supernatant, suggesting that they could be secreted forms (Robson et al., 2006; Zimmermann et al., 2012).

All these enzymes conserve five “apyrase conserved regions”, known as ACR1 to ACR5 (Robson et al., 2006; Zimmermann et al., 2012). They are usually located on the cell membrane with the catalytic site oriented to the extracellular milieu. Some of them are also bound to the organelle membrane, with the catalytic site oriented to the lumen of the organelle (Bernardes et al., 2000; Meyer-Fernandes, 2002; Sansom et al., 2008). The E-NTPDases are proteins characterized by having a high degree of glycosylation. Not only are there different numbers of N-glycosylation sites but also the placement of the sites relative to the apyrase conserved regions is different as well. For example, four out of seven glycosylation sites in NTPDase 3 are near the ACR regions, whereas NTPDase 4 and 5 have no glycosylation sites approximate to ACR regions (Zhong et al., 2017).

In parasites, nucleotidases/E-NTPDases seem to be involved in crucial processes, such as virulence, infectivity, purine salvage pathways, and parasite adhesion on the host cell (Sansom, 2012; Figueiredo et al., 2016; Paes-Vieira et al., 2018; Silva-Gomes et al., 2020). During parasite infection, the host cell releases ATP as a danger signal, leading to the augmentation of the extracellular ATP level (Trautmann, 2009). For instance, during *Leishmania* spp. infection, the parasite interacts with Toll-like receptors (TLR) present in the surface of the host cell, inducing the release of ATP to the extracellular milieu *via* pannexin-1 channels. The extracellular ATP can be now metabolized by parasite E-NTPDase and 5’-NT, increasing the extracellular Adenosine (Ado) level (Chaves et al., 2021). Such sequential hydrolysis of extracellular ATP to adenosine was demonstrated in different parasite species, indicating that not only E-NTPDases but also ecto-5`-nucleotidases could be present in protozoa parasites, including *Leishmania* spp., *Trypanosoma* spp.*, Trichomonas vaginalis, Tritrichomonas foetus, Toxoplasma gondii, and Schistosoma* spp. (Berrêdo-Pinho et al., 2001; Meyer-Fernandes, 2002; Sansom, 2012; Pimentel et al., 2016; Paes-Vieira et al., 2018).

Many studies have suggested the involvement of parasite E-NTPDases in host immune defense suppression since their activity would lead to the reduction of ATP and ADP levels. According to these studies, parasite E-NTPDases (CD39 gene family) hydrolyze extracellular ATP to ADP and AMP, which is, in sequence, hydrolyzed by the parasite ecto-5’-nucleotidases (CD73 gene family). The hydrolysis of AMP by parasite ecto-5’-nucleotidases increases the levels of adenosine, leading to the reduction of the host inflammatory response (Fredholm et al., 2007; Sansom et al., 2008; Arora and Rai, 2019).

Parasite E-NTPDases and 5’NTs have received special attention because they are cell-surfaced enzymes whose activity can modulate the host purinergic signaling, a signal transduction pathway mediated by purine nucleotides and nucleosides, such as ATP and adenosine, which bind and activate specific surface proteins called purinergic receptors (Figueiredo et al., 2016; Zimmermann 2021b). In host cells, two families of purinergic receptors, P1 and P2, mediate purinergic signaling. The P1 receptors are metabotropic G protein-coupled receptors, encompass four subtypes, called A_1_, A_2A_, A_2B,_ and A_3_; all of them use adenosine as ligand. The P2 receptors are responsive to tri- and diphosphonucleotides (ATP, ADP, UTP and UDP) and are divided into P2X and P2Y subtypes. The P2X receptor is also called ionotropic and functions as non-selective cation ion channel. Meanwhile, P2Y receptor is coupled to G protein. The family of P2Y receptors can modulate many cellular processes related to inflammatory and immune responses, such as cell differentiation, adhesion, migration, phagocytosis, and secretion in the host cell (such as macrophages, monocytes, neutrophils, and dendritic cells) (Coutinho-Silva and Savio, 2021; Klaver and Thurnher, 2021 **)**. The most well-studied of the P2X receptors is the P2X7 subtype. It has been shown that the interaction between ATP and the P2X7 receptor leads to an inflammatory response by the host immune system. Such inflammatory response triggered by extracellular ATP activates macrophage and dendritic cells, leading to an increase of IL-12 and TNF-α, reactive oxygen species (ROS), and nitric oxide (NO) production, culminating in intracellular pathogen elimination (Savio and Coutinho-Silva, 2019; Chaves et al., 2021; Coutinho-Silva and Savio, 2021).

**Figure 1:**
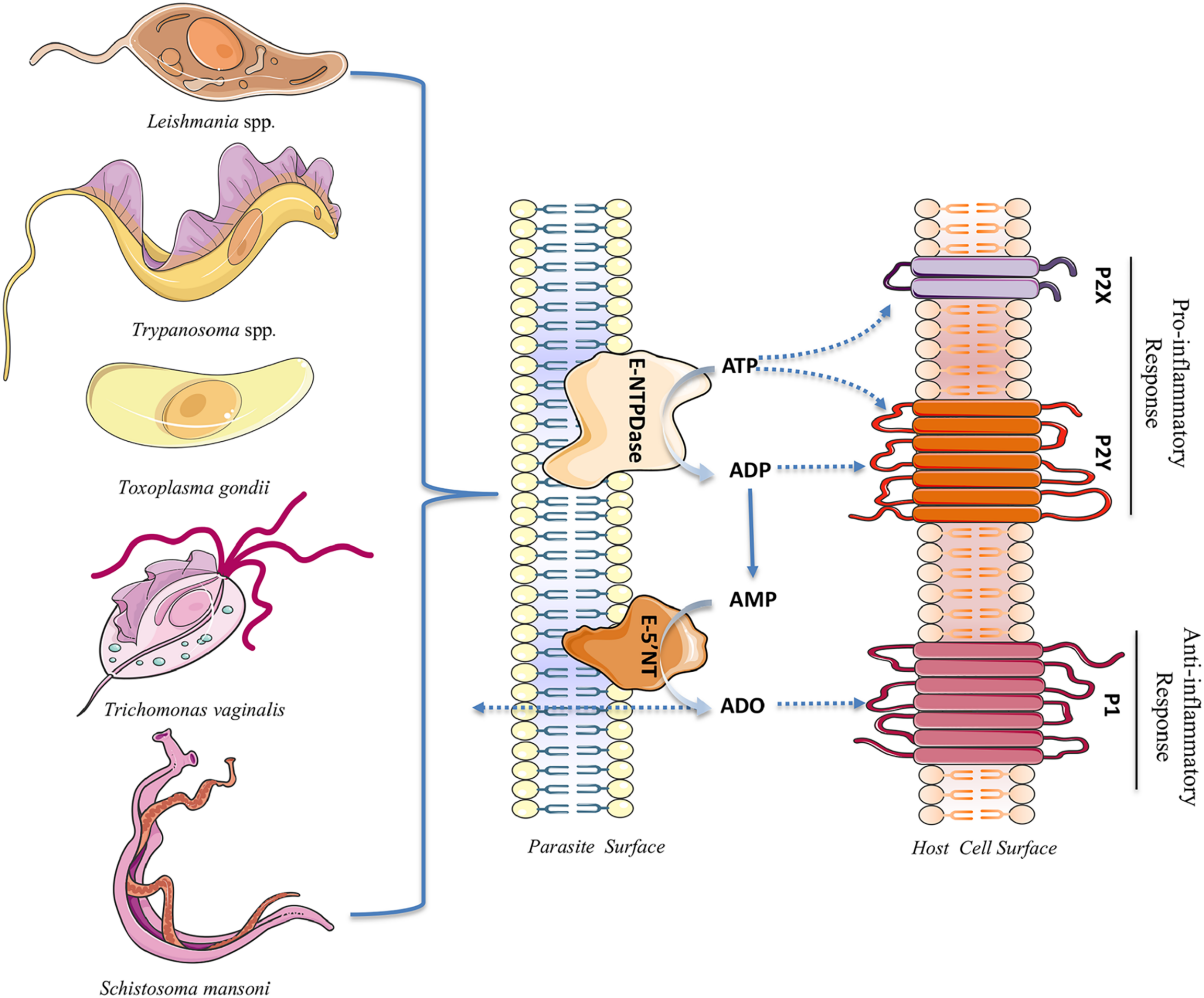
Extracellular nucleotidases in parasite cell surface and nucleotide/purinergic signaling in the host cell. During infection, the immune system interprets an increase in the levels of extracellular ATP as a risk situation and gives rise to an inflammatory response through the activation of P2X and P2Y receptors, culminating in parasitism control. In the parasite surface the enzyme NTPDase hydrolyzes extracellular ATP to ADP and ADP to AMP. The AMP generated can be subsequently hydrolyzed to adenosine (ADO) by Ecto-5’-nucleotidases (E-5’NT). Adenosine reduces the host inflammatory response *via* P1 receptors to favor the persistence of parasite in the infected cells. Changes in extracellular nucleotide and adenosine levels can modulate P2 and P1 receptors activity (the images used in the illustration are provided by https://smart.servier.com).

Acting on P1 receptors of the host cell, extracellular adenosine, generated by parasite E-NTPDases and 5’-NTs, increases the intracellular level of cAMP in the host cell, leading to the modulation of the inflammatory response (Figueiredo et al., 2017). In summary, adenosine leads to the suppression of inflammatory cytokines by macrophages and dendritic cells and the production of antimicrobial substances by macrophages and neutrophils. In addition, adenosine increases the level of IL-10, an important regulatory cytokine (Wilson et al., 2009; Figueiredo et al., 2016; Haskó et al., 2018; Antonioli et al., 2019). Thus, the dual effect of both ATP and adenosine on the host immune response seems to depend on different factors such as ATP and adenosine concentration, time of exposure, and conditions of the host cell. Thus, the balance between these two molecules (ATP and adenosine) is crucial for the proper immune response (Zhao et al., 2017). **Figure 1** summarizes the role/effect of parasite ectonucleotidases on the nucleotide/purinergic signaling of the host cell.

In this review article, we discuss the role of nucleotidases/E-NTPDases in parasite infectivity and virulence and show the potential of these enzymes as antiparasitic drug targets. In this sense, we also provide an overview of the latest studies concerning the development of nucleotidase/E-NTPDase inhibitors and their effect on parasite physiology.

## Development of E-NTPDases Inhibitors

Ecto-nucleotidases have been implicated in different pathological conditions. The interplay of ecto-nucleotidases with the nucleotide and adenosine receptor systems has received special attention. P1 and P2 receptor activity can be controlled by changes in the extracellular levels of both adenosine and nucleotide (Kukulski et al., 2011). Thus, the search for strong and subtype-specific ecto-nucleotidase inhibitors is important not only for developing potential therapeutics but also for developing proper molecular tools for studying the role of ecto-nucleotidases in parasite-host interaction. However, the development of such inhibitors is an obstacle to overcome since they must be E-NTPDase-specific, not affecting other types of ecto-nucleotidases or nucleotide receptors (Zimmermann, 2021a; Zimmermann, 2021b).

While several non-selective chemical compounds have been used to inhibit E-NTPDases activity, strong and specific inhibitors are rare, most of them are nucleotide analogs (Zimmermann 2021a; Zimmermann, 2021b). The most well-known CD39 inhibitors include an impermeant inhibitor, DIDS (4,4′-diisothiocyanostilbene-2,2′-disulfonic acid) (Meyer-Fernandes et al., 1997; Bernardes et al., 2000), sodium azide (NaN3), suramin (Bisaggio et al., 2003; Fonseca et al., 2006), chelators (EDTA and EGTA) (Iqbal and Shah, 2018), ARL67156 (6-N, N-diethyl-D-b,g-dibromomethylene ATP) (Lévesque et al., 2007), 8-BuS-ATP derivatives (8-thiobutyladenosine 50-triphosphate) (Gendron et al., 2000; Lecka et al., 2013) and BG0136 (1-naphthol-3, 6-disulfonic acid) (Gendron et al., 2002). Such inhibitors can increase ATP and ADP levels, causing the augmentation of purinergic signaling. However, inhibitors like suramin bind to P2 receptors and antagonize their effects (Munkonda et al., 2007). Since these chemicals are not CD39-specific, their use has been shown to be limited (Battastini et al., 2021). Other classes have been reported as inhibitors of E-NTPDases, such as polyoxometalates (POMs) (Lee et al., 2015; Schachter et al., 2015; Jeffrey et al., 2020), thiadiazolopyrimidones (Afzal et al., 2020), Schiff bases of tryptamine (Kanwal et al., 2019), quinoline (Hayat et al., 2019; Murtaza et al., 2021) and anthraquinone derivatives (Al-Rashida and Iqbal, 2014; Baqi et al., 2020), sulfopolysaccharides (Lopez et al., 2021) and carboxamide derivatives (Afzal et al., 2021). Moreover, specific antibodies have also been used as inhibitors of NTPDases (Munkonda et al., 2009; Pelletier et al., 2017).

Since E-NTPDases have been implicated in different processes of parasite physiology, such as differentiation, nutrition, invasion, survival in the host cell, and modulation of the host immune response to establish infection, these enzymes have been seen as promising targets for drug development (Paes-Vieira et al., 2018). In this sense, many works have shown the effects of several compounds on parasite E-NTPDases (Knowles, 2011; Pimentel et al., 2016; de Carvalho et al., 2019; da Silva et al., 2021).

### *Leishmania* spp.

The genus *Leishmania* encompasses at least 20 different species of protozoa parasites, which can cause a tropical disease called leishmaniasis. An estimated 0.7-1 million new cases of leishmaniasis per year are reported from nearly 100 endemic countries (Burza et al., 2018). Leishmaniasis is characterized by three different clinical manifestations, which are determined by the *Leishmania* species. Self-healing lesions in the infectious site characterize the cutaneous leishmaniasis (CL). In the mucocutaneous leishmaniasis (ML), besides cutaneous lesions, mucosal damage may occur, leading to disseminated or diffuse CL. In visceral leishmaniasis (VL), parasites migrate to the liver, spleen, and bone marrow, causing a more severe condition that can be fatal (Pace, 2014).

In *Leishmania* species (*L. amazonensis, L. mexicana, L. tropica, L. braziliensis, L. donovani, L. infantum, L. major)*, two genes encoding putative NTPDases (*L*NTPDase 1 and *L*NTPDase 2) have been found in genome databases (Sansom, 2012; Paes-Vieira et al., 2018). In different *Leishmania* species, *L*NTPDase and ecto-5’-nucleotidase activities have been implicated with virulence and infectivity (Berrêdo-Pinho et al., 2001; Pinheiro et al., 2006; de Almeida Marques-da-Silva et al., 2008; Souza et al., 2011; Leite et al., 2012; Maia et al., 2013).

In *L. amazonensis*, *La*NTPDase activity is differentially regulated among promastigote, amastigotes, and metacyclic promastigote forms. The authors showed that *La*NTPDase activity is lower in promastigotes, and significantly higher in metacyclic promastigote forms (Paes-Vieira et al., 2021). Moreover, overexpression of ntpd1 and ntpd2 led to a reduced lesion in mice transfected with such overexpressing-*L amazonensis*, compared to control. It was suggested that *L. amazonensis* overexpressing ntpd1 and ntpd2 leads to the augmentation of extracellular adenosine levels, modulating the host immune response and promoting pathogen clearance (Paes-Vieira et al., 2021). A mutant of *L. major* lacking the Golgi NTPDase 1 exhibited a delayed capacity to induce lesions in susceptible mice by promastigote forms. On the other hand, parasites not expressing the secreted *Lm*NTPDase 2 did not show alterations in *L. major* virulence (Sansom et al., 2014).

Interaction and infection of macrophage with *L. amazonensis* and *L. infantum* pretreated with anti-NTPDase antibodies were significantly reduced, suggesting that parasite E-NTPDases could be involved in host cell interaction and invasion (Pinheiro et al., 2006; Vasconcellos et al., 2014; Peres et al., 2018). In addition, the involvement of ecto-ATPases in the invasion process of macrophage by *L. amazonensis* was investigated in the works of Moreira et al. (2009) and Ennes-Vidal et al. (2011). The authors pretreated promastigote forms with an ecto-ATPase inhibitor known as CrATP (chromium (III) adenosine 5-triphosphate) and observed a decrement in the adhesion and endocytic indices. Polyclonal antibodies against a conserved B domain from the potato apyrase polypeptide (r-potDomain B) and synthetic peptides designed from the B domain (LbB1LJ and LbB2LJ) were able to reduce parasite NTPDase 1 activity in different species of *Leishmania* (Porcino et al., 2012; Detoni et al., 2013; Maia et al., 2013). These studies could contribute to the development of specific NTPDase inhibitors.

In *L. amazonensis*, extracellular adenosine (produced by sequential dephosphorylation of ATP by *La*NTPDases and ecto-5′-nucleotidase) binds to P1 receptors (A_2A_ and A_2B_) on macrophage membrane (Csóka et al., 2008; Figueiredo et al., 2012; Figueiredo et al., 2016). Because of the interaction between adenosine and P1 receptors, activated macrophages reduce IL-12 and TNF-α cytokines levels, leading to the decrement of nitric oxide (NO^•^) production and the establishment of parasites in the host cell. Thus, the increase of adenosine levels during *Leishmania* infection seems to contribute to the host-parasite interaction, increasing the parasitism and delaying the lesion remission (Olivier et al., 2005; Gomes et al., 2015; Vijayamahantesh et al., 2017). Interestingly, when the adenosine receptor is blocked or ablated, or even when adenosine is removed by enzymatic activity, during the infection, the host-parasite interaction is significantly reduced (Figueiredo et al., 2017).

The incubation of *L. amazonensis* (original strain) with DIDS (4,4’- diisothiocyanatostilbene 2,2’-disulfonic acid), a well-known *La*NTPDase inhibitor, prevents the reduction of NO^•^ by activated macrophages (J774), decreasing the parasite survival (Gomes et al., 2015). Moreover, the production of IL-12 and TNF-α by macrophages infected with DIDS-treated *L. amazonensis* was significantly higher than that observed for macrophages infected with untreated parasites. Such results suggest that the inhibition of the *La*NTPDase activity by DIDS reduced *L. amazonensis* capacity to down-modulate the release of inflammatory cytokines, which are essential for NO^•^ production by inducible Nitric Oxide Synthase (iNOS) (Gomes et al., 2015). The results obtained by Gomes et al. (2015) reinforce the role of *La*NTPDase in modulating the host immune system, which is crucial for the success of *L. amazonensis* infection.

Gomes et al. (2015) showed that the blockage of receptors A_2B_ in J774 macrophages led to the increase of NO^•^ production and the reduction of parasite survival in stimulated macrophages. The authors also showed that the blockage of A_2B_ receptors led to the increase of TNF-α and IL-12 production in macrophages infected with *L. amazonensis*, indicating the importance of adenosine production to the macrophages’ modulation. Interestingly, using the adenosine analog known as NECA (5’-(N-Ethylcarbox- amido) adenosine-NECA) during the infection of macrophages with the avirulent clone 1IIId, the authors observed an increase in the ability of the avirulent clone to survive within macrophages. Moreover, the treatment with NECA was able to reduce the levels of TNF-α, IL-12, and NO^•^ in macrophages, showing that the interaction between adenosine and A_2B_ receptors is essential for the host immune system modulation since the treatment with NECA was able to recover the capacity of the 1IIId clone to survive in stimulated macrophages. Thus, Gomes et al. (2015) described a mechanism that correlates *La*NTPDase/nucleotidase activity and the capacity of the parasite to down-modulate the host immune system. Specifically, the augmentation of adenosine levels at the beginning of infection seems to compromise the macrophage activation, leading to the blockage of cytokines on the host cell (Gomes et al., 2015).

Working with *L. amazonensis* promastigotes resistant to vinblastine (a cell division blocker), Giarola et al. (2014) observed an increase in the ecto-ATPase activity and a more severe disease scenario. The authors also found a two-fold higher ecto-ATPase protein expression in vinblastine-resistant promastigotes than in control cells. The data obtained by Giarola et al. (2014) show that there seems to be a positive correlation between higher ecto-ATPase activity and greater infectivity/disease severity.

### *Trypanosoma* spp.

Parasites from the genus *Trypanosoma* (Trypanosomatida, Kinetoplastea) are flagellated protozoa that cause a wide range of diseases in both humans and animals. Such parasites are transmitted between hosts by insect vectors (Hutchinson and Stevens, 2018). Among the numerous parasite species belonging to the genus *Trypanosoma*, some of them have medical (*T. cruzi*, which cause Chagas disease in the Americas and *T. brucei rhodesiense* and *T*. *brucei* *gambiense* that cause sleeping sickness in human African) and veterinarian (*T. evansi, T. vivax, T. brucei*, *T. copemani, T. equiperdum*) importance (Büscher et al., 2017).

*Trypanosoma cruzi* is the etiological agent of American trypanosomiasis or Chagas disease. The disease is endemic in the southern USA and 21 countries across Latin America, with ∼7 million people infected and 70 million at risk (Moretti et al., 2020). *Trypanosoma evansi* is the etiologic agent of surra, a disease that occurs in several animal species, such as equids, leading to significant losses in global production since it can be fatal when is late diagnosed (Desquesnes et al., 2013).

In *T. cruzi*, only one gene coding for *Tc*NTPDase is present in its genome, named *Tc*NTPDase 1 (Fietto et al., 2004; da Silva et al., 2021). The *Tc*NTPDase 1 coding sequence has been cloned and expressed in the bacterial system. Biochemical characterization showed that *Tc*NTPDase 1 is an Apyrase/CD39/E-NTPDase family member. Moreover, *Tc*NTPDase1 activity on *T. cruzi* membrane has also been demonstrated (Bisaggio et al., 2003; Fietto et al., 2004; Meyer-Fernandes et al., 2004; Santos et al., 2009; Giarola et al., 2013; Mariotini-Moura et al., 2014; da Silva et al., 2021).

Recently, Silva-Gomes et al. (2020) engineered *T. cruzi* parasites overexpressing the *Tc*NTPDase 1 gene and observed higher infectivity rates when compared to the control cell line. On the other hand, when *Tc*NTPDase 1 gene was silenced, the infectivity rates decreased significantly. These data are in agreement with the idea that *Tc*NTPDase 1 could be involved in *T. cruzi* infectivity (Silva-Gomes et al., 2020).

*In vitro* studies showed that *T. cruzi* adhesion and internalization into macrophages were significantly compromised when parasites were treated with DIDS and Suramin, a treatment that also inhibited the ecto-ATPase activity. On the other hand, the augmentation of ecto-ATPase activity was accompanied by the increase of parasite adhesion to macrophages (Bisaggio et al., 2003). Experiments using recombinant TcNTPDase 1 (acting as a competitor) or anti-*Tc*NTPDase 1 polyclonal antibodies (acting as blockers) evidenced the involvement of *Tc*NTPDase 1 in parasite-host interaction and cell adhesion (Mariotini-Moura et al., 2014).

Santos et al. (2009) also showed the importance of *T. cruzi* NTPDase 1 in infectivity and virulence. *T. cruzi* treated with E-NTPDase partial inhibitors (ARL67156, Gadolinium, and Suramin), and *Tc*NTPDase 1 antibody markedly reduced trypomastigotes infectivity. The authors observed lower levels of parasitemia when *Tc*NTPDase-inhibited trypomastigotes were used to infect mice, which increased the host survival (Santos et al., 2009).

Studies have shown that suramin is a potent inhibitor of *T. cruzi* E-NTPDase, and *in vivo* experiments suggest that this drug can negatively affect the purinergic signaling of host cells infected with *T. cruzi* (Bisaggio et al., 2003; Santos et al., 2009). Novaes et al. (2018) studied the effect of suramin in the chemotherapy of murine model of Chagas disease. *T. cruzi*-infected mice submitted to Suramin-based chemotherapy showed increased reactive tissue damage, inflammation, and parasitism. In addition, Suramin-based treatment aggravated myocarditis cases and increased the mortality rates (Novaes et al., 2018). This unexpected result could be explained by the fact that Santos et al. (2009) used Suramin only in the parasites, before infection, while Novaes et al. (2018) used Suramin in the host cells. In addition, it is worth mentioning that Suramin is a P2 antagonist in mammalian cells (Lambrecht et al., 2002). Thus, the apparent disagreement between the works of Santos et al. (2009) and Novaes et al. (2018) seems due to the absence of specificity of Suramin, acting on both P2 receptors and *T. cruzi* E-NTPDase. These results reinforce the importance of working with specific E-NTPDase inhibitors, whose absence has become an obstacle to be overcome (da Silva et al., 2021).

The enzyme adenosine deaminase (ADA) is responsible for regulating the extracellular adenosine concentration, converting adenosine to inosine (Franco et al., 1997). do Carmo et al. (2017) performed an experimental combined treatment of Chagas disease using both deoxyconformycin (a potent ADA inhibitor) and 3`-deoxyadenosine (adenosine analogue). According to the authors, the results observed in the experiments, which include reduced parasitemia and cardiac inflammatory infiltrates, are due to the modulation of seric NTPDase and ADA activities (do Carmo et al., 2017). Similar results were observed when mice were infected with *T. evansi* (Dalla Rosa et al., 2013; Dalla Rosa et al., 2015) and *T. brucei* (Rottenberg et al., 2005).

Fracasso et al. (2021) investigated the effects of benznidazole (BNZ, currently used in the treatment of Chagas Disease), and resveratrol (RSV, a natural polyphenol with antioxidant and neuroprotector activities) alone and in combination during acute *T. cruzi* infection in the mouse cerebral cortex. The treatment with RSV in the infected group reduced NTPDase and 5’-NT activity and diminished ATP, ADP, and AMP hydrolysis compared to the non-treated group. The combination of RSV + BNZ decreased AMP hydrolysis in infected animals compared to the non-treated group, exerting an anti-inflammatory effect. Treatment with BNZ and RSV alone or associated reduced adenosine (ADO) levels and converted inosine by E-ADA (adenosine deaminase extracellular). These results suggested a suitable immunosuppressive effect of RSV during acute *T. cruzi* infection. Thus, the authors concluded that resveratrol could act as a neuroprotective molecule, probably preventing inflammatory changes caused by infection by *T. cruzi*, even though the mice experienced high levels of parasitemia (Fracasso et al., 2021).

The pre-treatment with curcumin in rats infected with *T. evansi* decreased parasitemia and mortality (Wolkmer et al., 2013; Wolkmer et al., 2019), maintained the NTPDase activity reduced and enhanced ADA activity on the surface of lymphocytes. These results suggest that curcumin treatment can improve immunomodulatory response mediated by ecto-nucleotidases, favoring the response against the parasite (Wolkmer et al., 2019).

Taking together, these studies show that purinergic signaling carries out a pivotal function in the immune and inflammatory responses in the course of parasite infections.

### Trichomonas vaginalis

The flagellated protozoan *Trichomonas vaginalis* infects the human urogenital tract causing trichomoniasis, a sexually transmitted disease. In women, such infection can lead to cervical cancers, infertility, pelvic inflammatory disease, and pregnancy complications (Mielczarek and Blaszkowska, 2016).

Five putative NTPDase genes (*Tv*NTPDase1–5) were found in the *T. vaginalis* genome database (Frasson et al., 2016). Regarding substrate specificity, *T. vaginalis* NTPDase activity was able to hydrolyze both purine and pyrimidine nucleosides, 5-di- and 5-triphosphate (de Aguiar Matos et al., 2001; Frasson et al., 2012). In addition, *Tv*NTPDases localization and activity have been shown on *T. vaginalis* surface (de Jesus et al., 2002; Tasca et al., 2004), as well as on *T. foetus* surface (Jesus et al., 2002). The presence of such enzymes on the cell surface is important for parasite viability when exposed to extracellular nucleotides. Cell surfaced-localized *Tv*NTPDases play an important role concerning the maintenance of extracellular adenosine levels and the purine salvage pathway in the parasite (de Jesus et al., 2002; Jesus et al., 2002; Ferla and Tasca, 2021).

The effect of the compounds metronidazole (MTZ) and tinidazole (TNZ) on *T. vaginalis* NTPDase and 5’NT activities was investigated (Tasca et al., 2003). In this study, two strains were used, one long-term-grown isolate and one fresh clinical isolate. The hydrolysis of ATP and ADP was found to be 5- to 7-fold higher in the fresh clinical isolate than in the long-term-grown strain. In fresh clinical isolate, the compound TNZ inhibited the ATP hydrolysis by 33%. On the other hand, the same enzyme activity was induced by MTZ in long-term grown strain. When both isolates were treated with MTZ for 2 h, the ATP and ADP hydrolysis was significantly inhibited, while only fresh clinical isolate had the ATP and ADP hydrolysis inhibited by treatment with TNZ. The 5’NT activity was not changed by TNZ or MTZ treatment. Taken together, these data indicate that the parasite modulates the extracellular ATP and ADP levels when submitted to the treatment with TNZ and MTZ as a protection mechanism to survive in unfavorable conditions (Tasca et al., 2003; Ferla and Tasca, 2021).

The ecto-NTPDase activity of the *T. vaginalis* was inhibited by the plant alkaloids lycorine and candimine (Giordani et al., 2010; Petró-Silveira et al., 2020). Recent studies demonstrated that the effect of lycorine involves the inhibition of *T. vaginalis* NTPDase, generating extracellular ATP accumulation and stimulating ROS release by neutrophils. According to Petró-Silveira et al. (2020), lycorine could make *T. vaginalis* more susceptible to the host immune system, leading to parasite elimination. Moreover, during *T. vaginalis* infection, NO production by neutrophils, *via* A_2A_ receptors activation, seems to be dependent on both the ecto-5′-nucleotidase activity and the adenosine levels (Frasson et al., 2012). In addition, the levels of ROS and IL-8 were decreased by Adenosine in neutrophils infected with *T. vaginalis*, probably due to the activation of A1 adenosine receptors (Frasson et al., 2017). Thus, *Tv*NTPDAse and 5’NT activities carry out an important role in adenosine production, triggering an efficient purinergic-signaling cascade, which allows the parasite to escape the host immune response (Petró-Silveira et al., 2020). In addition, the treatment of trophozoites of *T. vaginalis* freshly isolated with hormones, especially steroids, also inhibited *Tv*NTPDase activity, and this inhibition seems to be associated with the decreased gene expression of the *Tv*NTPDases (Rückert et al., 2009; Rückert et al., 2010).

### Toxoplasma gondii

The intracellular protozoan *Toxoplasma gondii* infects mammals and other animals, causing a disease known as toxoplasmosis, which can be severe to the fetus, when infection takes place during pregnancy, and to immunocompromised individuals (Montoya and Liesenfeld, 2004).

Both isoforms, *Tg*NTPDase 1 and *Tg*NTPDase 2 are expressed in virulent strains, while in avirulent strains only the *Tg*NTPDase 2 isoform is expressed. These isoforms have different enzymatic specificity. *Tg*NTPDase 1 hydrolyzes nucleoside triphosphate, and *Tg*NTPDase 2 hydrolyzes diphosphate substrates. However, both forms of *Tg*NTPDase are activated by dithiothreitol (Asai et al., 1995; Nakaar et al., 1998).

The *T. gondii* NTPDases have become a target in drug development. Parasites expressing an antisense *Tg*NTPDase-RNA showed reduced *Tg*NTPDase expression and activity. In addition, parasites expressing the antisense *Tg*NTPDase-RNA showed a lower ability to proliferate, suggesting that *Tg*NTPDases could be involved in *T. gondii* replication (Nakaar et al., 1999). This conclusion was supported by studies with monoclonal antibodies against NTPDase 2 of *T. gondii* that inhibited *Tg*NTPDase activity and decreased *T. gondii* tachyzoites replication (Tan et al., 2010).

The treatment of mice infected with *T. gondii* with Diphenyl diselenide (PhSe)2 reduced histological inflammatory markers, ROS levels, and ADA activity in the spleen, besides reversing splenomegaly. In addition, *Tg*NTPDase and 5’NT activity were increased in the spleen, stimulating the hydrolysis of ATP and the enhancement of adenosine production in the tissue. These results indicate that purinergic signaling could be involved in the pathogenesis of *T. gondii* infection (Doleski et al., 2017a; Doleski et al., 2017b).

Bottari et al. (2020) demonstrated that mice treated with resveratrol during chronic infection by *T. gondii* increased NTPDase and reduced ADA activities. The adenosine signalization by regulating A_1_ and A_2A_ receptors expression promoted anti-inflammatory and neuroprotector mechanisms during *T. gondii* infection (Bottari et al., 2020).

### *Schistosoma* spp.

The genus Schistosoma holds helminth parasites responsible for causing a human disease known as Schistosomiasis, which causes liver, intestinal, lung, and chronic pelvic inflammations (Colley and Secor, 2014).

In *Schistosoma mansoni* two NTPDase isoforms (*Sm*ATPDases 1 and 2) were detected and partially purified. It was shown that *S. mansoni* NTPDase activity can be found throughout the parasite life cycle, besides the involvement in the worm metabolism and the host-parasite interaction (Vasconcelos et al., 1993; Vasconcelos et al., 1996; DeMarco et al., 2003; Da'dara et al., 2014). *Sm*ATPDase 1 is located on the surface and *Sm*ATPDase 2 is in the tegument and secreted by eggs and adult worms (Faria-Pinto et al., 2004; Fietto et al., 2004; Levano-Garcia et al., 2007). Both isoforms showed immune cross-reactivity with antipotato apyrase antibodies (Faria-Pinto et al., 2008; Faria-Pinto et al., 2010). Macrophages incubated with antibodies against the ATPDase isoforms of *S. mansoni* showed reduced NTPDase 1 activity, and the parasite proliferation was inhibited (Marconato et al., 2017).

*Schistosoma* eggs found in the host tissues trigger an inflammatory response known as granulomatous, characterized by the presence of granulomas in the infected tissues (Farah et al., 2000). Gusmão et al. (2021) showed that immunization of C57BL/6 mice with potato apyrase was ineffective in triggering a protective immune response against *Schistosoma* infection. Differently, immunization using potato apyrase decreased the granulomatous response, accompanied by an enhancement of multinucleated giant cells. In this sense, immunotherapies developed from potato apyrase-immunization to avoid granuloma development seems to be a potential prophylaxis strategy (Gusmão et al., 2021).

Recently, studies have demonstrated that some flavonoids and chalcones (natural plant metabolites), such as licoflavone B and cardamonin display antiparasitic activities against adult worms of *S. mansoni* (de Carvalho et al., 2015; de Castro et al., 2015; Souza et al., 2017). The effects of licoflavone B and cardamonin on the *S. mansoni* NTPDase activity were investigated. In addition, a molecular docking approach was also used to study the mode of action of such compounds on *S. mansoni* ATPDase 1 (de Castro et al., 2015; de Carvalho et al., 2015). de Castro et al. (2015) showed that ATP-diphosphohydrolase activity was inhibited by about 82% by cardamonin (40 μM), with an IC_50_ of 23.54 μM, while ADPase activity was unchanged. In addition, interactions between cardamonin and *Sm*ATPase1 seem to be done especially through hydrogen and hydrophobic bonds, as revealed by molecular docking studies (de Castro et al., 2015). Regarding licoflavone B, a concentration of 40 μM was able to abolish 84% of the ATP-diphosphohydrolase activity, with an IC_50_ of 23.78 μM. Different from cardamonin that did not show *Sm*ADPase activity inhibition, licoflavone B in a concentration of 40 μM inhibited 56% of the *Sm*ADPase activity. The interaction between licoflavone B and *Sm*ATPase 1 seems to involve nine dock positions at the Nucleotide-Binding Site of the enzyme (de Carvalho et al., 2015; de Carvalho et al., 2019).

The schistosomicidal effect of two synthetic chalcones (*E*-4’-hydroxychalcone and *E*-2’-hydroxy-4-methoxychalcone) was studied by Pereira et al. (2018). The authors showed that both compounds can inhibit *S. mansoni* ATP-diphosphohydrolase activity, besides causing a decrease in parasite load in mice. Martins et al. (2000) evaluated the inhibitory effect of sesquiterpene lactones on *S. mansoni* ATP-diphosphohydrolase. The authors showed that the compounds thapsigargin and thapsigargicin inhibited 70% and 57% of the enzyme’s activity, respectively.

## Concluding Remarks

Many studies have described the involvement of E-NTPDase activity in parasite nutrition, differentiation, invasion, survival in the host cell, modulation of the host immune response, and establishment of infection. Since E-NTPDases have been implicated in important events for different parasite species, such enzymes have been considered as ideal drug targets for many therapeutic applications, including parasitic diseases. In addition, ecto-nucleotidase activity associated with the purinergic signaling seems to be crucial to parasite-host interaction, besides contributing to adenine acquisition *via* the purine salvage pathway.

In this context, this review emphasizes the importance of ecto-nucleotidases in parasite physiology and pathogenesis. Moreover, we highlight the importance of such enzymes as drug target candidates to the development of new antiparasitic compounds, including those able to inhibit specifically the different ecto-nucleotidases isoforms.

Although many compounds have been assayed against ecto-nucleotidases from different parasites to inhibit the enzyme activity, the low specificity of these molecules represents an obstacle to be eliminated, still requiring huge efforts in the search for new selective and effective ecto-nucleotidase inhibitors.

## Author Contributions

LP-V, AG-V, and JM-F designed the study and wrote the paper. LP-V, AG-V, and JM-F edited and reviewed the final version of the manuscript. All authors contributed equally to the article and approved the submitted version.

## Funding

This work was supported by grants from the Brazilian funding agencies Conselho Nacional de Desenvolvimento Científico e Tecnológico (CNPq - Grant Number: 401134/2014–8), Coordenação de Aperfeiçoamento de Pessoal de Nível superior (CAPES - Grant Number: 0012017), Fundação Carlos Chagas Filho de Amparo à Pesquisa do Estado do Rio de Janeiro (FAPERJ - Grant Number: e-26/201.300/2014) to JM-F.

## Conflict of Interest

The authors declare that the research was conducted in the absence of any commercial or financial relationships that could be construed as a potential conflict of interest.

## Publisher’s Note

All claims expressed in this article are solely those of the authors and do not necessarily represent those of their affiliated organizations, or those of the publisher, the editors and the reviewers. Any product that may be evaluated in this article, or claim that may be made by its manufacturer, is not guaranteed or endorsed by the publisher.
